# Simplification of culture conditions and feeder-free expansion of bovine embryonic stem cells

**DOI:** 10.1038/s41598-021-90422-0

**Published:** 2021-05-26

**Authors:** Delia Alba Soto, Micaela Navarro, Canbin Zheng, Michelle Margaret Halstead, Chuan Zhou, Carly Guiltinan, Jun Wu, Pablo Juan Ross

**Affiliations:** 1 Department of Animal Science, University of California, 450 Bioletti Way, Davis, CA 95616 USA; 2 Instituto de Investigaciones Biotecnológicas ‘Dr Rodolfo Ugalde’, UNSAM-CONICET, Buenos Aires, Argentina; 3 Department of Molecular Biology, University of Texas Southwestern Medical Center, 5323 Harry Hines Boulevard, Dallas, TX 75390 USA; 4 Hamon Center for Regenerative Science and Medicine, University of Texas Southwestern Medical Center, 5323 Harry Hines Boulevard, Dallas, TX 75390 USA

**Keywords:** Embryonic stem cells, Pluripotent stem cells

## Abstract

Bovine embryonic stem cells (bESCs) extend the lifespan of the transient pluripotent bovine inner cell mass in vitro. After years of research, derivation of stable bESCs was only recently reported. Although successful, bESC culture relies on complex culture conditions that require a custom-made base medium and mouse embryonic fibroblasts (MEF) feeders, limiting the widespread use of bESCs. We report here simplified bESC culture conditions based on replacing custom base medium with a commercially available alternative and eliminating the need for MEF feeders by using a chemically-defined substrate. bESC lines were cultured and derived using a base medium consisting of N2B27 supplements and 1% BSA (NBFR-bESCs). Newly derived bESC lines were easy to establish, simple to propagate and stable after long-term culture. These cells expressed pluripotency markers and actively proliferated for more than 35 passages while maintaining normal karyotype and the ability to differentiate into derivatives of all three germ lineages in embryoid bodies and teratomas. In addition, NBFR-bESCs grew for multiple passages in a feeder-free culture system based on vitronectin and Activin A medium supplementation while maintaining pluripotency. Simplified conditions will facilitate the use of bESCs for gene editing applications and pluripotency and lineage commitment studies.

## Introduction

Embryonic stem cells (ESCs) are derived from the inner cell mass (ICM) of preimplantation embryos and capture indefinitely the developmental potency of the transient pluripotent epiblast. ESCs were first established in mice^1, 2^, subsequently in rhesus macaque^3^, humans^4^, rats^5, 6^, and recently in cattle^7–9^ and pigs^10, 11^. Mouse ESCs have revolutionized developmental biology by enabling complex genetic modifications to be passed on to the progeny^12, 13^. Stable livestock ESCs represent a promising tool for genomic selection, producing gametes in vitro, developing complex genetic modifications valuable for agriculture and biomedicine, and for understanding cell fate decisions during early development^14, 15^. After years of research and many attempts to establish pluripotent stem cells (PSCs) from ungulate species^15^, stable bovine PSCs were only recently reported^7–9^. Primed pluripotent bESC lines were efficiently established from in vitro produced bovine blastocysts in a condition that resembles the culture of human region-selective PSCs^16^. The combined inhibition of Wnt/tankyrases by IWR-1, stimulation of FGF2 signaling, use of mitotically-inactivated MEF, and use of custom-made base medium similar to mTeSR1 but devoid of TGFβ1^17^, allowed derivation of stable bESC lines^7^. Even though the reported culture system consistently supported bESC growth in an undifferentiated state for multiple passages, custom-made mTeSR1 base medium is cumbersome to prepare and not readily available from a commercial source.

Cell culture medium formulation and preparation is labor-intensive, time consuming and prone to batch-to-batch inconsistencies, making it impractical for research-scale cell production^17, 18^. Custom-made mTeSR1 base medium consists of a mixture of more than 50 components, thus a consistent and readily available base medium alternative is necessary to simplify and standardize bESC culture.

Mitotically inactivated feeder cells were first used in 1955 to support the clonal expansion of HeLa cells^19^ and the subsequent establishment of PSCs lines has expanded their use. Today, MEF feeder cells are widely used to culture PSCs from different species. MEF feeder cells are proliferation-arrested and metabolically-active, and secrete into the culture medium a still-not-completely-elucidated mixture of cytokines and growth factors^20, 21^. MEF feeder cells also play a role in detoxifying the culture medium and acting as a substrate to favor cell attachment^22^. However, the undefined nature of MEFs limits reproducibility and scale-up, and complicates mechanistic studies. In attempts to replace MEF feeders, synthetic coatings, synthetic biomolecules, and recombinant proteins have been used to culture PSCs. Human vitronectin, a defined supporting substrate alternative, has allowed derivation and expansion of human PSCs under chemically defined feeder-free culture conditions^18, 23^. Recombinant fibronectin has also shown to support proliferation of mouse and human PSCs, and the recently derived bovine expanded potential stem cells (EPSCs)^7–9^. However, it remains unknown if bESCs can be adapted to other feeder-independent culture conditions that support their pluripotency.

A PSC culture system that relies on a complex medium formulation and MEF feeder cells is not ideal for a broad and routine use of bESCs. Therefore, we sought to simplify bESCs derivation and culture to replace custom-made base medium by a commercially available alternative and eliminate the need for MEF feeders. Here we report the successful establishment of bESC lines in a commercially available culture medium base and their stable expansion under a feeder-free condition.

## Results

### NBFR culture condition enables derivation of stable bESC lines

Based on similarities in their composition, we tested N2B27^16, 24^ and Essential 6^18^ commercially available defined culture media for substitution of complex mTeSR1 base medium. We initially adapted CTFR-bESCs^7^ (Custom-made mTeSR1 devoid of TGFβ1 and supplemented with FGF2 and IWR-1) to Essential 6 and N2B27 base media, supplemented with FGF2 and IWR-1 for 4–6 passages. While cells grown in Essential 6 base medium progressively degenerated, those cultured in N2B27 base medium survived adaptation, maintaining proliferative growth rate and expression of pluripotency factor SOX2; however, heterogenous expression of OCT4 was observed (Fig. S1). Next, we evaluated the effect of supplementing Essential 6 and N2B27 conditions with different concentrations of KSR or BSA on self-renewal and pluripotency. CTFR-bESCs cultured in Essential 6 medium containing 5% or 10% KSR lost typical colony morphology and progressively stopped proliferating. Supplementation of Essential 6 medium with 0.1% or 0.5% BSA improved bESC colony morphology and proliferation rate, but cells still presented heterogeneous expression of pluripotency marker OCT4 (Fig. S2). CTFR-bESCs adapted to N2B27 medium supplemented with 5% or 10% KSR maintained proliferation rate but grew in non-compact colonies with undefined borders, making the identification of ESCs among MEF feeders difficult. Instead, when N2B27 medium was supplemented with 0.1% or 0.5% BSA, cells grew in well-defined compact colonies, maintaining proliferation rate and homogenous expression of pluripotency factors OCT4 and SOX2 (Fig. S2). The higher BSA concentration (0.5%) resulted in more homogenous expression of pluripotency markers and larger colonies. Together, these results indicate that N2B27 base medium supplemented with BSA supports self-renewal of bESCs. Considering that higher BSA concentration improved pluripotency marker expression, colony size, and to better resemble the high BSA content (~ 1.3%) in mTeSR1 medium^17^, we increased BSA concentration to 1% for further experiments. Overall, we optimized a new culture condition for expansion of bovine bESC based on commercially available N2B27 medium supplemented with 1% BSA, 20 ng/mL FGF2 and 2.5 µM IWR-1, which we termed NBFR.

**Figure 1:**
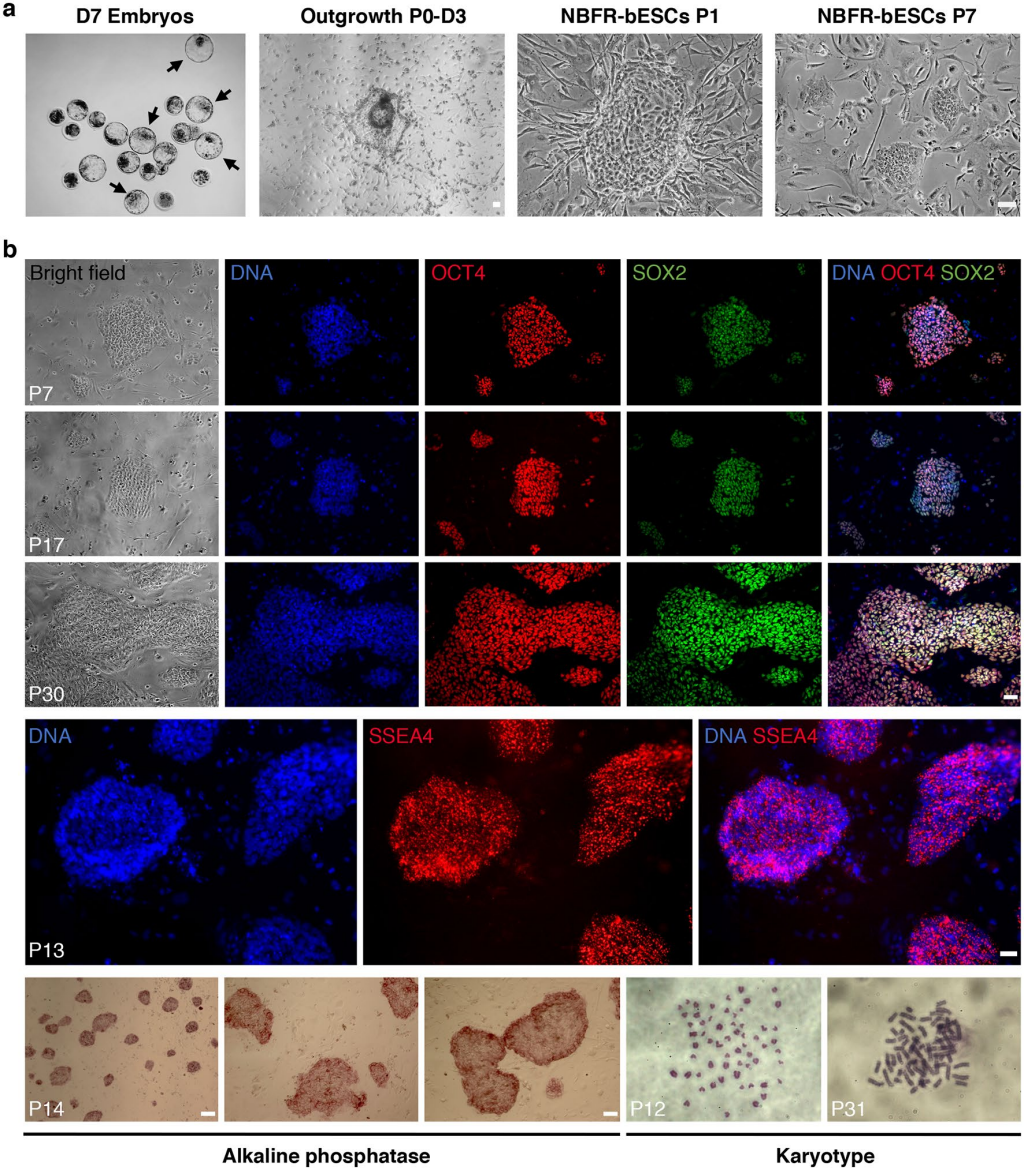
Derivation and characterization of bESCs in NBFR conditions. (**a**) Bright field images at different stages of bESC derivation from whole blastocysts. Images represent day-7 embryos selected for ESC derivation (arrows), outgrowth after 3 days in culture, and typical colony morphology at passages 1 and 7. (**b**) Immunofluorescence staining of OCT4 and SOX2 pluripotency factors, and SSEA4 surface marker. Detection of alkaline phosphatase activity, and chromosome number (2n = 60) of bESCs at different passages. D: day; P: passage. Scale bars 100 μm.

Using NBFR culture conditions, we derived new bESC lines (NBFR-bESCs) from whole blastocysts or isolated ICMs plated on MEF feeders (Figs. 1a and S3a). Out of 28 whole single blastocysts, 7 pools of 3–4 whole blastocysts, and 7 isolated ICMs, 10 bESC lines were established (8, 1, and 1, respectively), representing 4 female and 6 male lines; indicating that bESCs can be established from both whole blastocysts and isolated ICMs, as previously reported for CTFR conditions^7^. NBFR-bESCs grew for multiple passages (> 35) in well-defined colonies, were easily subcultured by single-cell dissociation, presented positive alkaline phosphatase activity, and maintained a stable karyotype (2n = 60). Immunofluorescence analysis indicated that NBFR-bESCs consistently expressed OCT4 and SOX2 pluripotency markers upon long-term culture (Fig. 1b), and lacked expression of CDX2 and GATA6, trophectoderm (TE) and primitive endoderm (PE) markers, respectively (Fig. S3b). NBFR-bESCs were positive for SSEA4 staining (Fig. 1b), but negative for SSEA1, TRA-1-60 and TRA-1-81 (Fig. S3c). Quantitative RT-PCR confirmed expression of pluripotency factors *OCT4*, *SOX2,* and *NANOG* in NBFR-bESC lines (NBFR-bESCs #A and #D), and the absence of *CDX2* (TE), *FOXA2* (endoderm), *MEOX1* (mesoderm), and *PAX6* (ectoderm) lineage specific markers, which were instead specifically expressed in representative tissues and whole blastocysts (Fig. 3a). Overall, gene marker expression indicates that NBFR cells present typical pluripotency features.

**Figure 2:**
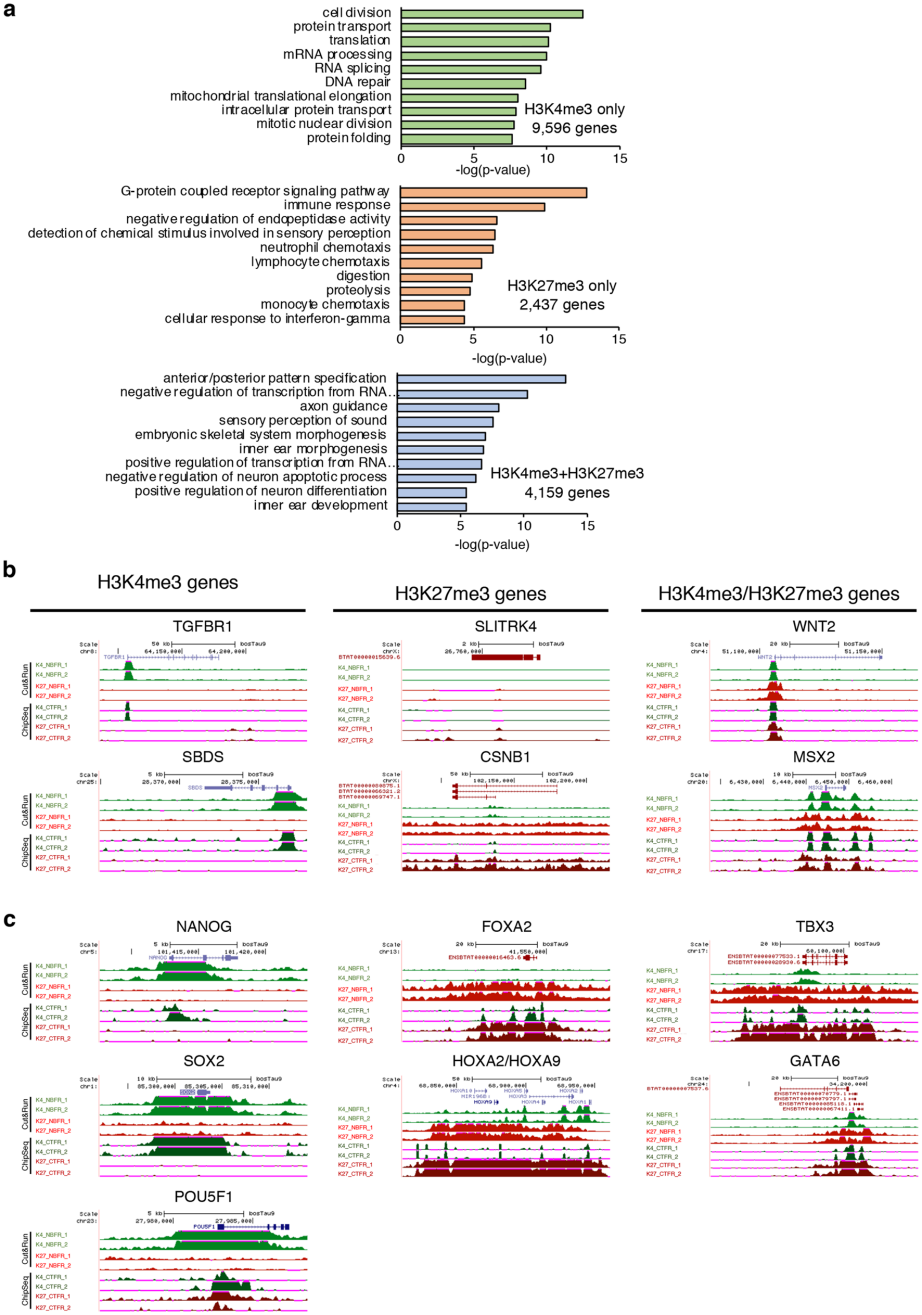
Epigenomic landscape of NBFR-bESCs. (**a**) Functional enrichment of genes containing H3K4me3, H3K27me3 or bivalent domains. The top 10 GO terms are shown. (**b**) Track view of genes containing H3K4me3, H3K27me3 or bivalent H3K4me3/H3K27me3 domains. (**c**) Track view of H3K4me3 and H3K27me3 profiles in primed and naïve pluripotency markers. Two independent NBFR lines (NBFR1 and NBFR2) were analyzed by CUT&RUN for H3K4me3 (K4) and H3K27me3 (K27) and compared with Chip-Seq data from CTFR-bESC7 (CTFR1 and CTFR2).

To define the histone methylation profile of NBFR-bESCs we performed Cleavage Under Targets and Release Using Nuclease (CUT&RUN)^25^ using antibodies against H3K4me3 and H3K27me3; which we compared to published ChIP-seq results from CTFR-bESCs^7^. Globally, NBFR-bESCs had similar epigenetic characteristics to CTFR-bESCs (Fig. 2). Among protein-coding genes, 9,596 genes contained only H3K4me3, 2,437 genes contained only H3K27me3, and 4,159 genes were bivalent, containing both H3K4me3 and H3K27me3 (Fig. 2a, Table S2). Similar to CTFR-bESCs, gene ontology (GO) revealed that NBFR-bESC had H3K4me3 enrichment in genes related to cellular homeostasis. Genes that showed H3K27me3 enrichment (e.g., *CSNB1*, *SLITRK4*) were associated with functions that are usually downregulated in pluripotent stem cells; while bivalent domains were mostly observed in genes related to cell fate decisions (e.g., *WNT2* and *MSX2*; Fig. 2b)^26^. To further characterize the pluripotency state of NBFR-bESC lines, we investigated the epigenetic profile of genes that are commonly related to naïve or primed states. H3K4me3 was found in *POU5F1*, *SOX2*, *NANOG* and *SALL4* genes, which is a common epigenetic pattern from both naïve and primed states^26^. However, similar to CTFR-bESCs, the accumulation of H3K27me3 in *HOXA9* gene as well as the presence of bivalents domains in *HOXA1*, *FOXA2*, *GATA6*, and *TBX3* genes indicated that NBFR-bESC lines also displayed epigenetic features typical of the primed state (Fig. 2c)^26^. All together, we concluded that NBFR culture medium supports derivation and self-renewal of pluripotent bESCs with molecular similarities to CTFR-bESCs and the primed pluripotency state.

### NBFR-bESCs undergo multilineage commitment in vitro and in vivo

**Figure 3:**
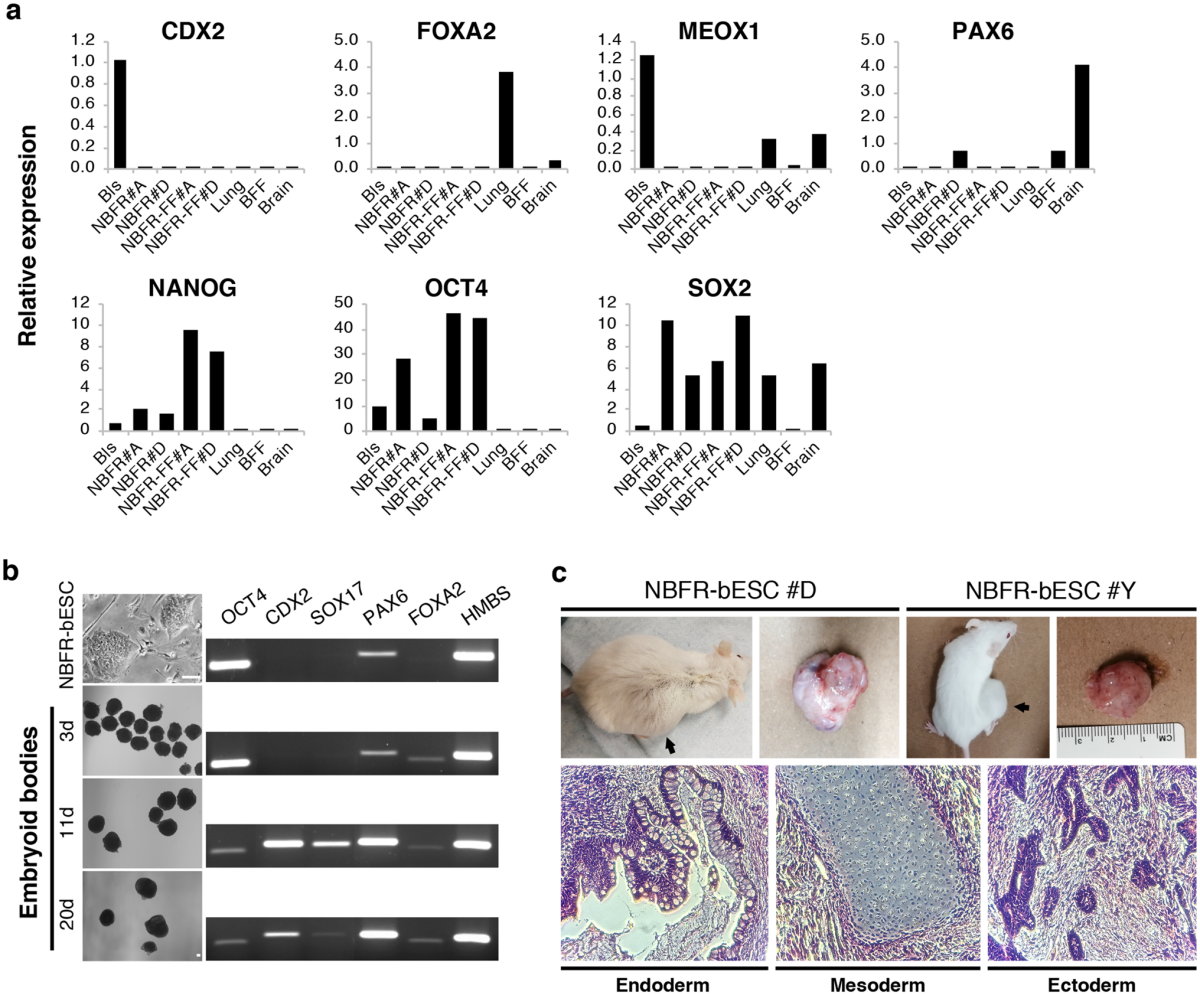
In vitro and in vivo differentiation of NBFR-bESCs. (**a**) Gene expression levels of pluripotency (*OCT4*, *SOX2*, *NANOG*), trophectoderm (*CDX2*), endoderm (*FOXA2*), ectoderm (*PAX6*), and mesoderm (*MEOX1*) markers in representative tissues and two independent NBFR-bESCs lines before (NBFR#A and NBFR#D at passage 10) and after adaptation to feeder-free culture (NBFR-FF #A and #D, passage 10 and 20 after adaptation, respectively). Relative expression was calculated using the comparative CT method (ΔΔCT), normalizing values to *HMBS*. (**b**) Bright field images of NBFR-bESCs and embryoid bodies obtained at different times of differentiation. RT-PCR for genes representative of different lineages (*OCT4*, *CDX2*, *SOX17*, *PAX6,* and *FOXA2*). *HMBS*: housekeeping gene. Scale bar 100 μm. (**c**) Teratomas obtained 12 weeks after injection of two independent NBFR-bESC lines (NBFR #D and #Y) into immunodeficient mouse. Representative histological images showing derivates of the 3 germ lineages. BFF: bovine fetal fibroblasts; Bls: blastocysts, d: days.

Pluripotency of NBFR-bESCs was evaluated by formation of embryoid bodies (EBs) and teratomas. After three weeks of differentiation as EBs, endodermal (*FOXA2* and *SOX17*), mesodermal (*CDX2*), and ectodermal genes (*PAX6*) were progressively upregulated (Fig. 3b). For teratoma evaluation, one male (NBFR-bESCs #D) and one female (NBFR-bESCs #Y) cell lines were injected into immunodeficient mice. After 12 weeks, each cell line resulted in the formation of one teratoma similar in size (~ 2 cm in diameter) containing tissues representative of the three germ layers (Fig. 3c). These results indicate that NBFR-bESCs have the capacity to differentiate into derivatives of the three germ lineages, thereby demonstrating their pluripotent status.

### NBFR-bESCs self-renew and maintain pluripotency in feeder-free conditions

We next examined the possibility of culturing NBFR-bESCs in feeder-independent conditions. We initially cultured CTFR-bESCs on vitronectin or Matrigel and found that regardless of the substrate, cells proliferated poorly and expressed OCT4 heterogeneously, which is a sign of differentiation (Fig. S4a). However, when culture medium was supplemented with Activin A (20 ng/mL), a growth factor known to support expansion of human ESCs (hESCs) under feeder-free conditions^27^, cell proliferation and self-renewal were maintained (Fig. S4b). On both substrate types, cells grew as a monolayer instead of forming colonies. Despite the different colony morphology in feeder-free conditions, CTFR-bESCs homogenously expressed OCT4 and SOX2 pluripotency markers (Fig. S4b). Considering the undefined nature of Matrigel, further assays were performed using vitronectin as a substrate.

**Figure 4:**
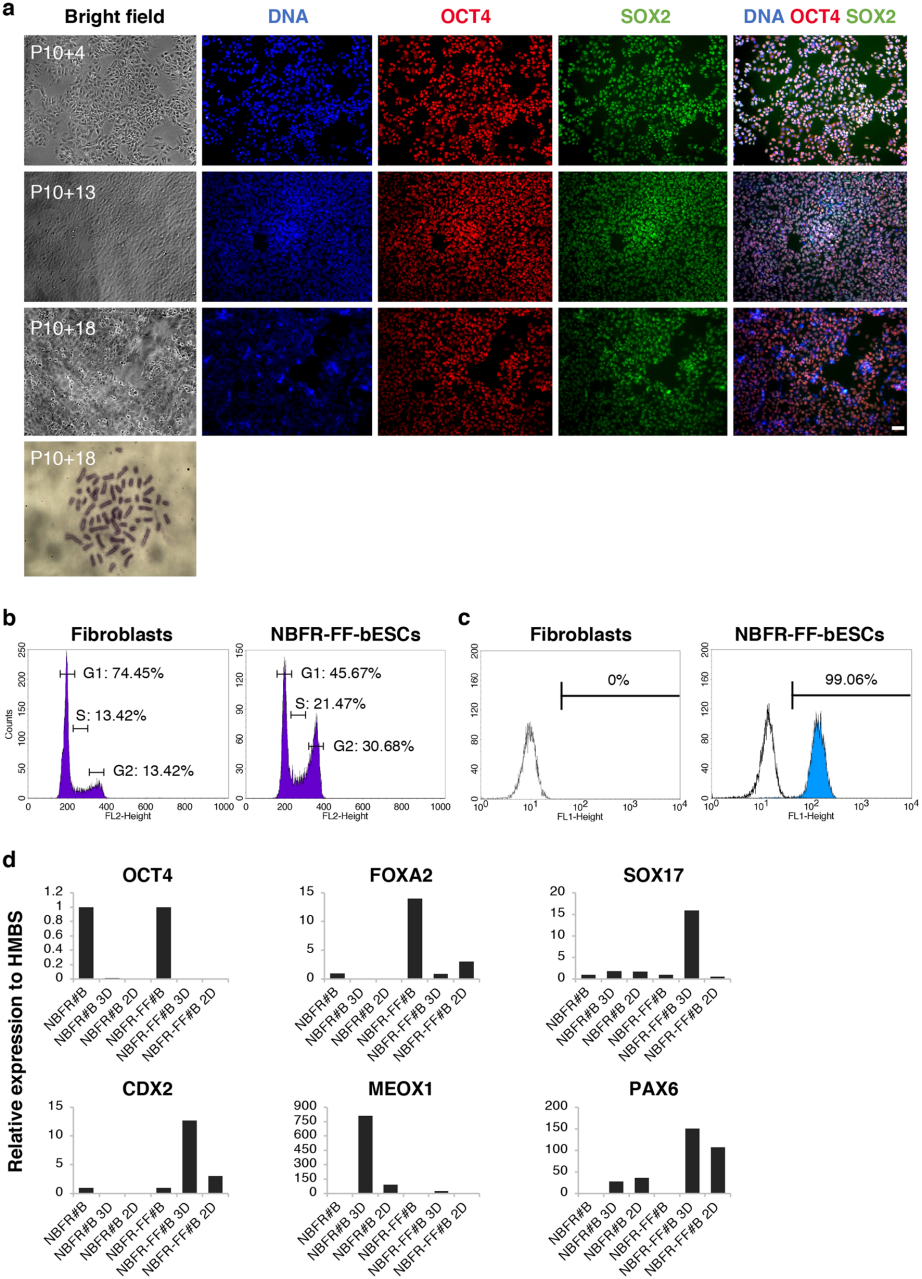
Characteristics of NBFR-bESCs grown under feeder-free conditions. (**a**) Immunostaining of OCT4 and SOX2, and normal chromosome number (2n = 60) of NBFR-bESCs grown for multiple passages on vitronectin supplemented with Activin A (**b**) Flow cytometry analysis of bovine fibroblasts and NBFR-bESCs cultured in feeder-free conditions (NBFR-FF-bESCs) indicating the DNA content and the percentage of cells in each phase of the cell cycle. (**c**) Quantification of OCT4 expression in NBFR-FF-bESCs (blue peak) and secondary only control (unshaded peak). (**d**) Expression levels of genes (*OCT4*, *FOXA2, SOX17, CDX2, MEOX1, PAX6*) representative of different lineages after 3D (3 weeks) and 2D (4 weeks) in vitro differentiation in NBFR-bESCs lines before (NBFR#B at passage 10) and after adaptation to feeder-free culture (NBFR-FF#B at passage 11). Relative expression was calculated using the comparative CT method (ΔΔCT), normalizing values to *HMBS*. P: passage. Scale bar 100 μm.

Next, we evaluated if feeder-independent conditions, which included vitronectin substrate and Activin A supplementation, would support expansion of NBFR-bESC lines. In agreement with our previous findings, NBFR-bESCs cultured in feeder-free conditions (NBFR-FF-bESCs) grew as a monolayer instead of forming colonies but regain their typical colony morphology when returned to culture on MEF feeders. Furthermore, NBFR-FF-bESCs maintained self-renewal and proliferated for more than 30 passages while showing stable expression of pluripotency markers and normal karyotype (Fig. 4a). Flow cytometry analysis of their DNA content showed that NBFR-FF-bESCs exhibited a shorter G1 phase of the cell cycle when compared to bovine fibroblasts (Fig. 4b), which is a characteristic of pluripotent cells undergoing rapid proliferation^7, 28^. Quantification of OCT4 expression by flow cytometry indicated that > 99% of NBFR-FF-bESCs were positive for the pluripotency factor (Fig. 4c). Additionally, quantitative RT-PCR of two different NBFR-FF-bESC lines (NBFR-FF #A and #D) showed that *OCT4*, *SOX2*, and *NANOG* where highly expressed in NBFR-FF-bESCs while *CDX2*, *MEOX1*, *FOXA2*, and *PAX6* were not detected, similar to NBFR-bESCs cultured on MEF feeders before adaptation to feeder-free conditions (NBFR #A and #D) (Fig. 3a). Upon in vitro differentiation, NBFR-FF-bESCs showed upregulation of genes representative of the three germ lineages (*CDX2*, *FOXA2, SOX17, MEOX1,* and *PAX6*), which indicates their potential for trilineage commitment (Fig. 4d). Overall, these results indicate that NBFR-bESCs retained self-renewal when adapted to feeder-free conditions, and that feeder-free culture supports propagation of bESCs for an extended period of time.

### Activin A and not IWR-1 sustains pluripotency of NBFR-FF-bESCs

**Figure 5:**
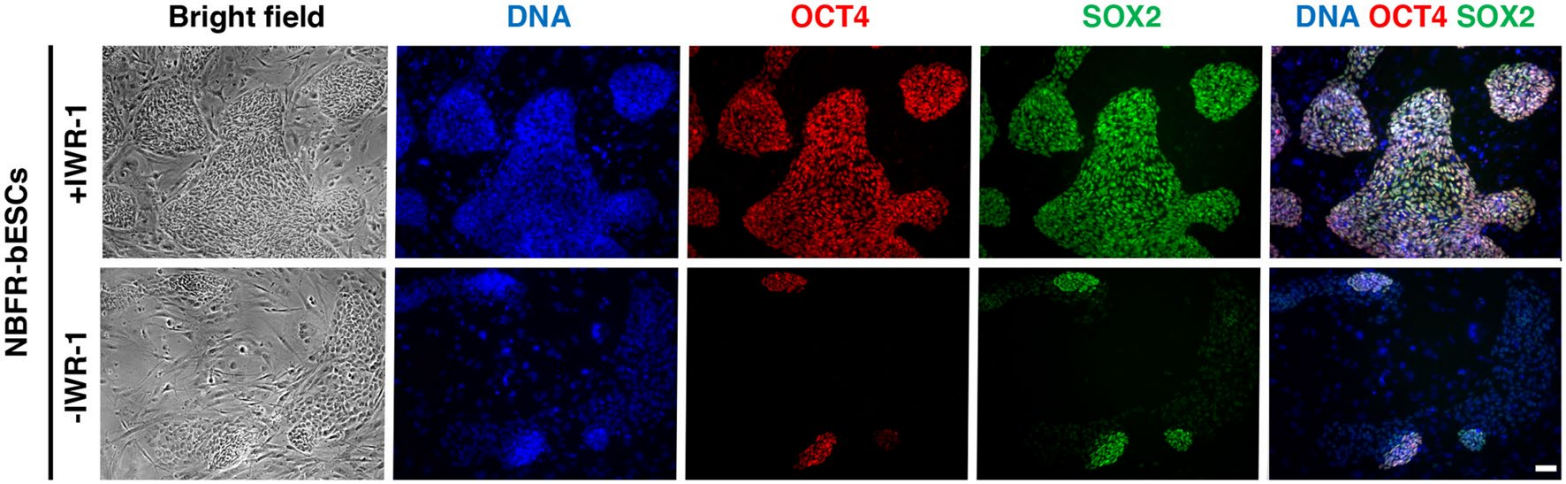
IWR-1 supplementation is required for pluripotency of NBFR-bESCs cultured on MEF feeders. Immunostaining of pluripotency factors OCT4 and SOX2 in NBFR-bESCs grown on MEF feeders for 5 passages after IWR-1 withdrawal. Scale bar 100 μm.

Self-renewal of bESCs relies on inhibition of Wnt/tankyrase signaling^7, 9^; therefore, we evaluated if NBFR-bESCs are also dependent on Wnt/tankyrase inhibition. After IWR-1 withdrawal from culture for 4 passages, immunofluorescence staining indicated loss of expression of OCT4 and SOX2 transcription factors (Fig. 5). Thus, inhibition of Wnt/tankyrase signaling is required for self-renewal of NBFR-bESCs and maintenance of their pluripotent state.

**Figure 6:**
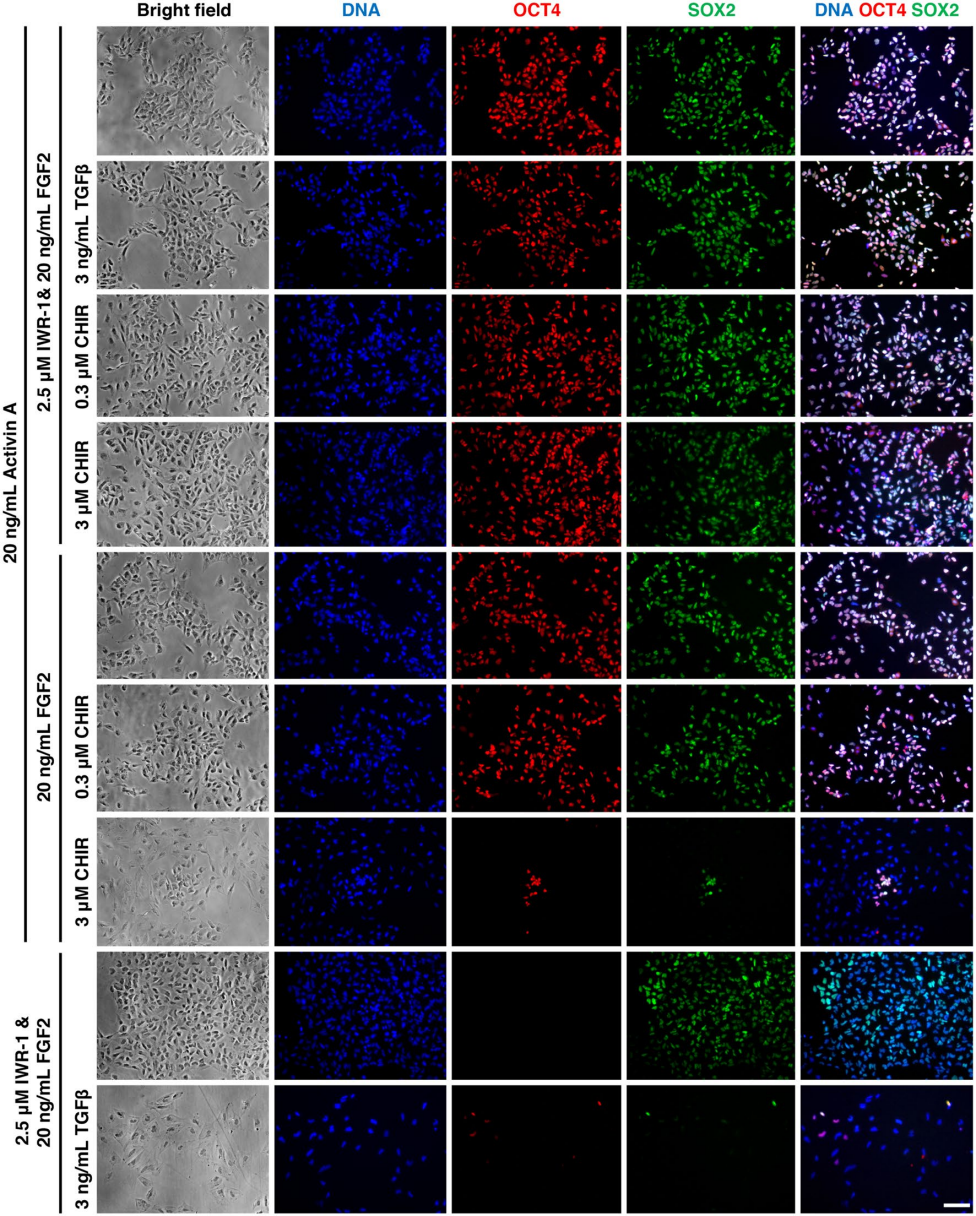
Feeder-free NBFR-bESCs depend on FGF2 and Activin A for pluripotency maintenance. Immunostaining of pluripotency factors OCT4 and SOX2 in NBFR-bESCs grown in feeder-free conditions supplemented with different combinations of Activin A, CHIR2291, FGF2, IWR-1 and TGFβ1 for 4 passages. Scale bar 100 μm.

Next, we evaluated the effect of different inhibitors and growth factors on NBFR-FF-bESCs, in terms of cell proliferation and expression of pluripotency factors (Fig. 6). After culturing cells for 5 passages, cells survived adaptation in all conditions evaluated except when FGF2 was removed from the culture medium. In the absence of FGF2, cells stopped proliferating and died after 2 passages. NBFR-FF-bESCs maintained expression of pluripotency genes and proliferation capacity in presence of 3 ng/mL TGFβ or 0.3 μM CHIR9921 supplemented to the medium. Surprisingly, withdrawal of IWR-1 from the culture medium did not have a detrimental effect on NBFR-FF-bESCs, with self-renewal being indistinguishable from control NBFR-FF-bESCs in presence of IWR-1. However, if CHIR9921 was included at a concentration of 3 μM in absence of IWR-1, NBFR-FF-bESCs lost expression of pluripotency markers OCT4 and SOX2, similar to IWR-1 withdrawal from NBFR-bESC on MEF feeders. Instead, in the presence of IWR-1, CHIR9921 at 3 μM had no effect on OCT4 and SOX2 expression. These results suggest that in the absence of Wnt activation, IWR-1 is indispensable for bESC pluripotency, and that MEF feeders may stimulate Wnt signaling, and thus IWR- is necessary for culturing bESCs on feeders^7^.

As described above, when Activin A was removed from the culture, NBFR-FF-bESCs lost expression of pluripotency markers and underwent differentiation. Additionally, even though Activin A and TGFβ belong to the same superfamily of morphogens and signal through the same downstream mediators^29^, at 3 ng/mL TGFβ1 did not rescue self-renewal of NBFR-FF-bESCs in the absence of Activin A. These results indicate that Activin A plays a crucial role in maintaining bESC pluripotency.

## Discussion

After years of efforts to isolate ESCs from bovine embryos, the recently reported CTFR culture system was the first to support the efficient derivation and stable long-term self-renewal of pluripotent bESCs with robust in vivo pluripotency capacity^7^. However, the potential applications of CTFR-bECSs are hindered by the undefined and complex nature of the CTFR culture system. Thus, the overarching goal of this study was to establish a chemically defined and simple bESC culture condition by employing commercially available components, facilitating medium preparation, and eliminating the need of MEF feeders for bESC expansion. Our results indicate that bESCs can be efficiently derived, and retain self-renewal and long-term pluripotency, when N2B27 base medium supplemented with BSA is used instead of mTeSR1 base medium. NBFR cells presented characteristics of primed bESCs, similar to previously described CTFR-bESCs. In addition, NBFR-bESCs could be grown for an extended period on vitronectin substrate (feeder-free) in the presence of Activin A while maintaining pluripotency features.

Custom mTeSR1 medium devoid of growth factors consists of DMEM/F12 plus a series of different supplements, among those BSA^17^. Therefore, it is not surprising that N2B27 base medium, which is also based on DMEM/F12^30^, supported long-term culture of bESCs after BSA supplementation. N2B27 has also been demonstrated to support the culture of PSCs in several other species, including cattle^8, 16, 24, 30^. Interestingly, even though Essential 6 is also based on DMEM/F12, it did not show the same potential for replacing custom mTeSR1, even after BSA supplementation. Besides DMEM/F12, Essential 6 and custom mTeSR1 media each consist of 5 and 17 other ingredients, respectively. Therefore, one or more of those 12 components, not included in Essential 6 medium (glutathione, L-Glutamine, defined lipids, thiamine, trace elements B, trace elements C, β-mercaptoethanol, BSA, pipecolic acid, LiCl, GABA, H_2_O), have roles on self-renewal and colony morphology of bESCs.

BSA functions as a carrier of lipids and metal ions, as an antioxidant, and has roles in the metabolic activity, proliferation and survival of cultured cells^31^. The exact mechanism of action of BSA in supporting PSCs has not yet been elucidated. Our results indicate that unlike BSA, KSR did not support self-renewal of bESCs. KSR contains a lipid-rich BSA, commercially known as AlbuMAX, which has been shown to be the active component supporting self-renewal of hESCs^32^. Instead, the BSA used in our media formulation contains very low levels of fatty acids, supporting the finding that KSR was not an equivalent alternative to BSA in bESC culture. On the other hand, it is known that BSA helps prevent the toxic effects of β-mercaptoethanol in hESCs culture^18^. We did not observe a toxic effect of β-mercaptoethanol in the absence of BSA when culturing bESCs in N2B27 medium, and the incorporation of BSA to Essential 6 medium, which does not contain β-mercaptoethanol in its formulation, improved survival of bESCs. Thus, our results suggest that the purified BSA protein used in our culture system is acting through a mechanism that is not just counteracting the toxic effect of β-mercaptoethanol. The role of BSA in bESC self-renewal and proliferation will have to be elucidated in order to find a chemically-defined substitute, especially considering that BSA is the only media component that is not completely defined.

Previously reported putative bovine PSCs have been largely characterized by detection of pluripotency and cell surface markers^15^. Even though most reports agree on the expression of pluripotency related transcription factors OCT4, SOX2, and NANOG, the expression of cell surface markers varies greatly between lines. Interestingly, NBFR-bESCs were positive for SSEA4, which is a typical marker of human PSCs, but instead NBFR-bESCs did not present SSEA-1, a typical marker of rodent PSCs; or TRA-1-60 and TRA-1-81, typically detected in human PSCs^4, 33, 34^. On the other hand, pig ESCs strongly expressed SSEA1, SSEA4, TRA-1-60 and TRA-1-81^11^, with pig EPSCs also expressing SSEA1 and SSEA4^10^. Contrary, bovine EPSCs showed only minimal signal after immunostaining against SSEA1 and SSEA4^8^. With large variation in cell surface marker expression between species and cell lines, it is difficult to ascertain the value of characterizing these markers in new, conversely, transcription factors have a clear functional role in maintaining pluripotency and are therefore highly valuable for characterizing pluripotent stem cells.

Chemically defined growth conditions are essential for many PSC applications, hence the undefined nature of the products secreted by MEF feeders limits the potential use of bESCs. Matrigel has consistently supported the feeder-free culture of human PSCs^17, 35–37^. Matrigel is a partially defined extracellular basement membrane matrix extracted from the Engelbreth-Holm-Swarm mouse sarcoma that contains a mixture of collagen type IV, laminin, and heparin sulfate proteoglycan^38, 39^. However, due to its partial definition, different defined extracellular matrix proteins have been tested for the culture and derivation of PSCs, where vitronectin has shown superior results, equivalent to Matrigel^18, 23^. In addition, recombinant fibronectin was recently used to support the expansion of bovine EPSCs while maintaining colony morphology and expression of alkaline phosphatase^9^. In this work, bESCs retained expression of pluripotency markers in feeder-free culture conditions that included vitronectin or Matrigel as a substrate. NBFR-FF-bESCs presented a high proliferation rate, conserved an euploid karyotype, maintained homogeneous pluripotency marker expression and were able to differentiate in vitro. Interestingly in both substrates, NBFR-FF-bESCs grew as monolayers instead of forming colonies, in contrast to what was observed after fibronectin adaptation of EPSCs. However, when NBFR-FF-bESCs were returned to culture on MEF feeders, they regained the typical colony morphology of NBFR-bESCs (data not shown). Overall, these results suggest that the cell substrate influences morphological aspects of bESCs without affecting their self-renewal.

Activin A is known to be essential for maintaining pluripotency of human and mouse PSCs by controlling expression of pluripotency factor NANOG, which in turn prevents neuroectoderm differentiation^40^. Prior evidence has associated the poor secretion of Activin A of non-mouse feeder cells, with inefficient maintenance of PSCs in an undifferentiated state^41^. Our results indicate that NBFR-bESCs require Activin A for self-renewal, and that the levels secreted by MEF feeders are not always sufficient to sustain long-term bESC growth. Thus, the addition of 20 ng/mL Activin A to the culture medium allowed long-term maintenance of bESCs cultured on MEF feeders or on vitronectin. Downstream ligand-specific receptors, Activin A and TGFβ exert their effect through the same SMAD2/3 proteins^29^. In the presence of FGF2, Activin A and TGFβ1 have the same effect maintaining long-term pluripotency and self-renewal of hESC^42, 43^. Thus, we evaluated the potential of TGFβ1 to substitute Activin A in the culture of NBFR-FF-bESCs. Interestingly, our results indicated that the role of Activin A in maintaining pluripotency of bESCs could not be replaced by 3 ng/mL of TGFβ1. Indeed, in bovine EPSCs the simultaneously supplementation of Activin A and TGFβ1 was necessary to maintain pluripotency. Therefore, more research will be needed to decipher the exact mechanism of Activin A and TGFβ1 signaling in self-renewal of bovine PSCs, and its resemblance with PSC of other species.

In agreement with previous reports^7, 9^, we observed that inhibition of Wnt signaling by IWR-1 and stimulation of FGF2 pathway are indispensable requirements to establish bESC lines. Similar to mouse and human PSCs^44, 45^, bESCs also rely on FGF2 for maintaining self-renewal and cell proliferation. Wnt signaling is involved in different cellular processes such as cell differentiation, cell migration, cell proliferation, and maintenance of pluripotency^46–51^. Members of the Wnt family are highly conserved secreted glycoproteins that activate different intracellular signaling cascades by binding to a repertoire of receptors^52^. Major downstream signaling branches include a canonical or Wnt/β-catenin dependent pathway as well as multiple non-canonical or β-catenin-independent pathways, which include the planar cell polarity and the Wnt/Ca^2+^ pathways^53^. Canonical Wnt signaling has been associated with lineage fate determination in bovine preimplantation embryos^54, 55^. Interestingly, the establishment of bovine PSCs relies on inhibition of Wnt pathway; instead, derivation of bovine trophoblast stem cells requires the opposite, activation of Wnt signaling through Wnt3a^56^. Inhibition of Wnt pathway or tankyrases by IWR-1, seems to be required in supporting pluripotency and blocking the expansion of extraembryonic cells during bESC line establishment on MEF feeders^7^. However, in absence of MEF feeders, inhibition of Wnt pathway was not necessary to sustain self-renewal of NBFR-FF-bESCs, suggesting that Wnt ligands that trigger bESC differentiation are secreted by the feeder cells; which MEF are known to do^47, 51^. We confirmed these hypotheses by mimicking the effect of Wnt differentiation cues by culturing NBFR-FF-bESCs in presence of 3 μM CHIR99021, a glycogen synthase kinase-3 (GSK-3) inhibitor that triggers activation of canonical Wnt signaling. Indeed, our findings indicated that in the presence IWR-1, CHIR99021 did not have an evident effect on bESCs, although those components were essential to derive bovine EPSCs^9^. However, if IWR-1 was withdrawn from the culture medium, CHIR99021 supplementation led to loss of pluripotency marker expression and subsequent cell differentiation. The role of Wnt in human ESC self-renewal remains controversial. Activation of canonical Wnt pathway by Wnt3a or BIO (6-bromoindirubin-3'-oxime), another GSK-3 inhibitor, was shown to sustain self-renewal in feeder-free culture conditions^47^. However, others concluded that stimulation of canonical Wnt pathway in hESCs leads to differentiation and does not support long-term pluripotency^57–59^. The conflicting roles of Wnt signaling in ESCs could be a result of incorrect experimental design^60^, incomplete understanding about the spectrum of activity of the inhibitors used, or the intricated relationships between different members of the Wnt signaling pathway. Wnt signaling participates in a wide variety of developmental and physiological processes, and because of the well-known cross-talk between its different branches, the exact role of Wnt signaling is difficult to determine. More research will be needed to clarify the mechanism underlining the bESC pluripotency program and to determine how well this process is conserved between livestock species and humans. Interestingly, inhibition of Wnt/tankyrase signaling was also shown to support establishment of PSCs from pig blastocysts^10, 11^.

Altogether, results from this study indicate that bESCs maintain long-term pluripotency when cultured in a simplified and mostly chemically defined culture system. Replacing custom-made base medium and MEF feeders with commercially available alternatives maintains the consistency of culture conditions and expands the potential uses of bESCs. A more amenable culture condition, especially one independent of feeder cells, could facilitate the use of bESC for complex gene editing, facilitating cell transfection and antibiotic resistance selection. A bESC culture system less prone to variations will also facilitate pluripotency and lineage commitment studies, such as the use of bESC for in vitro generation of meat or gametes. Our results also shed light into the mechanism involved in bovine pluripotency, which in absence of the unknown role of MEF feeders, is supported by Activin A and FGF2 signaling cascades in line with primed pluripotency of other species.

## Materials and methods

All experiments were performed in accordance with relevant guidelines and regulations.

### Adaptation of CTFR-bESC to different commercial base media

CTFR-bESC lines^7^ were cultured on MEF feeders (A34180, Gibco) in CTFR medium [custom mTeSR1^17^ growth factor-free supplemented with 20 ng/mL human FGF2 (100-18B, PeproTech) and 2.5 μM IWR-1 (I0161, Sigma)] at 37 °C and 5% CO_2_. CTFR-bESCs were adapted to N2B27^16^ and to commercially available Essential 6 (05947, Stemcell Technologies) base media for 4–6 passages. N2B27 medium consisted of a mixture of 1:1 DMEM/F12 medium (11320-033, Gibco) and Neurobasal medium (21103-049, Gibco), 0.5% v/v N-2 Supplement (17502-048, Gibco), 1% v/v B-27 Supplement (17504-044, Gibco), 2 mM MEM Non-Essential Amino Acid Solution (M7145, Sigma), 1% v/v GlutaMAX Supplement (35050-061, Gibco), 0.1 mM 2-mercaptoethanol (M6250, Sigma), 100 U/mL Penicillin, and 100 μg/mL Streptomycin (15140-122, Gibco). N2B27 and Essential 6 media were initially supplemented with 20 ng/mL human FGF2 and 2.5 μM IWR-1. In subsequent experiments, different levels of low fatty acid bovine serum albumin (0.1%, 0.5%, 1%) (BSA, 0219989950, MP Biomedicals) or knockout serum replacement (5%, 10%) (KSR, 10828-028, Gibco) were included in the media formulation. Adaptation of cells to new media alternatives was performed progressively on MEF feeders in two replicates. The first week of adaptation, CTFR-bESCs were grown in an equal mixture of CTFR, and N2B27 or Essential 6; subsequently CTFR medium was completely replaced.

### Derivation and culture of NBFR-bESCs

Bovine embryos (*Bos taurus*) were produced by in vitro fertilization of in vitro matured slaughterhouse-derived oocytes as previously reported^61^. In vitro cultured blastocyst stage embryos were collected seven days post fertilization. Unhatched blastocysts were treated with 2 mg/mL of Pronase (10165921001, Sigma) for 2–3 min to remove the zona pellucida and then thoroughly washed in SOF-HEPES to remove traces of the enzyme. In some experiments, ICMs were isolated from zona-free blastocysts by immunosurgery. Zona-free blastocysts were incubated in 20% bovine antiserum (B8270, Sigma) for 1 h at 38.5 °C and 5% CO_2_ followed by several washes in SOF-HEPES medium. Then, embryos were incubated in 20% guinea pig complement (S1639, Sigma) for 1 h at 38.5 °C and 5% CO_2_. Finally, blastocysts were washed in SOF-HEPES to gently remove trophectoderm cells by repeated pipetting. Whole zona-free blastocysts or isolated ICMs were placed on a monolayer of MEF feeders in separate wells of a 48-well dish previously coated with 0.1% gelatin (G9391, Sigma) and cultured in NBFR medium [N2B27 medium, 1% low fatty acid BSA, 20 ng/mL human FGF2, 2.5 μM IWR-1] supplemented with 10 μM Rho Kinase inhibitor Y-27632 (ROCKi, ALX-270-333, Enzo) and Antibiotic/Antimycotic Solution (100 U/mL Penicillin, 100 μg/mL Streptomycin 0.25 μg/mL Amphotericin B) (20004, JR Scientific), and incubated at 37 °C and 5% CO_2_. After 24 h, blastocysts that failed to adhere to the feeder layer were physically pressed against the bottom of the culture dish with a 22G needle to facilitate attachment. Thereafter, the culture medium was changed daily and supplementation with Antibiotic/Antimycotic Solution was used only during the first week of derivation. Every 6–7 days, outgrowths were dissociated and passaged using TrypLE Express (12604-013, Gibco), and re-seeded at a 1:1 split ratio in the presence of 10 μM ROCKi onto newly plated MEF. After 3–4 weeks in culture, bESC lines were considered established if cells grew homogeneously throughout the surface of the culture well in defined colonies. Once established, NBFR-bESC lines were grown at 37 °C and 5% CO_2_ on MEF feeders or adapted to vitronectin (A14700, Gibco) or matrigel (E1270, Sigma) substrates. Cells were sub-cultured every 3–4 days at a 1:5–1:10 split ratio. bESCs cultures required 20 ng/mL supplementation of Activin A (338-AC, R&D Systems) in absence of MEF feeders, or in feeder-dependent cultures beyond passage 20. To increase cell survival, bESC culture medium was supplemented with 10 μM ROCKi at seeding or cryopreservation. Culture media was changed daily. Blastocysts used for NBFR-bESC line derivation were obtained from 12 independent sessions of in vitro fertilization. All cell lines were checked for mycoplasma contamination once a month using LookOut Mycoplasma PCR Detection Kit (MP0035, Sigma) following manufacturer’s instructions.

The effect of different combinations of inhibitors and growth factors on bESCs was studied by culturing two independent feeder-free NBFR-bESCs lines for 4 to 5 passages in each media formulation. Medium formulation included 2.5 μM IWR-1, 0.3 μM and 3 μM CHIR99021 (SML1046, Sigma Aldrich), 20 ng/mL Activin A, 20 ng/mL FGF2 and 3 ng/mL TGFβ1 (HZ-1011, Proteintech).

### Immunofluorescence staining

NBFR-bESCs were grown to 80% confluency and fixed using fresh 4% paraformaldehyde (PFA, SC-281692, Santa Cruz Biotechnology) for 10 min at room temperature. After fixation, cells were permeabilized and blocked with 0.3% Triton-X100 (T8787, Sigma) and 3% v/v of Normal Donkey Serum (NDS, D9663, Sigma) in Dulbecco’s Phosphate-Buffered Saline (DPBS, 14040174, Gibco) for 30 min at room temperature. Cells were then incubated for 1 h at room temperature with the following primary antibodies: anti-POU5F1 (1:300; sc-8628, Santa Cruz Biotechnology), anti-SOX2 (1:300; NU579-UC, Biogenex), anti-CDX2 (1:300; MU392A-UC, Biogenex), anti-GATA6 (1:300; sc-9055; Santa Cruz Biotechnology), anti-SSEA1 (5 μg/mL; MC-480; Developmental Studies Hybridoma Bank), anti-SSEA4 (5 μg/mL; MC-813-70; Developmental Studies Hybridoma Bank), anti-TRA-1-60 (1:250; sc-21705; Santa Cruz Biotechnology) and anti-TRA-1-81 (1:250; sc-21706; Santa Cruz Biotechnology) in DPBS supplemented with 0.3% Triton-X100 and 1% v/v of NDS. Secondary antibody incubation was performed in the dark for 1 h at room temperature with 1:500 fluorescently labeled donkey anti-rabbit IgG Alexa Fluor 488 antibody (A-21206, Invitrogen), donkey anti-mouse IgG Alexa Fluor 568 antibody (A-21043, Invitrogen), donkey anti-goat IgG Alexa Fluor 568 antibody (A-11057, Invitrogen), and donkey anti-mouse IgG Cy3 antibody (715-165-151, Jackson ImmunoResearch). Hoechst 33342 (62249, Gibco) was used for counterstaining. Cells were observed in a Nikon TE2000-U inverted microscope and photographed using a Coolsnap EZ camera. Expression of pluripotency factors SOX2 and OCT4 was evaluated in all the established NBFR-bESC lines, whereas GATA6, CDX2, SSEA1, SSEA4, TRA-1-60, TRA-1-81 expression was evaluated in two independent NBFR-bESC lines.

### Detection of alkaline phosphatase activity

NBFR-bESCs lines were grown to 80% confluency and detection of alkaline phosphatase (AP) was performed using the Alkaline Phosphatase Staining Kit II (00-0055, Stemgent) following the manufacturer’s protocol. Briefly, bESCs were washed in DPBS with 0.05% Tween 20 (170-6531, Biorad) and fixed for 5 min at room temperature. Then, fixed cells were washed and incubated with freshly prepared AP Substrate Solution protected from light, at room temperature for 15 min, followed by a DPBS wash. After staining, cells were observed in a Nikon TE2000-U inverted microscope and photographed using a Research Instrument DC2 camera. AP activity was evaluated in all the established NBFR-bESC lines.

### Karyotyping

NBFR-bESCs grown to 50–60% confluency were incubated in fresh culture medium supplemented with 200 ng/mL Demecolcine (D7385, Sigma) for 1 h at 37 °C, 5% CO_2_. Cells were then harvested using TrypLE Express and incubated in KCl hypotonic solution (0.075 M) for 10 min at 37 °C. After incubation, cells were fixed in Carnoy’s fixative (3:1; methanol:acetic acid) for 15 min at room temperature, repeating 3 times. Fixed cells were resuspended in 100 μL of Carnoy’s fixative and the cell suspension was dropped onto a clean chilled slide. Slides were air-dried for 10–15 min and stained with 5% Giemsa stain (GS500, Sigma) for 10 min. Slides were rinsed in deionized water, air-dried, and mounted using ClearMount mounting medium (MMC0112, American MasterTech). Metaphase spreads were visualized at 100X magnification under oil immersion using a Nikon TE2000-U inverted microscope and photographed by a Research Instrument DC2 camera. Karyotype was studied in all the established NBFR-bESC lines.

### Flow cytometry analysis

Flow cytometry analysis was performed using the BD Cytofix/Cytoperm Fixation/Permeabilization Kit (554714, BD Biosciences). For quantification of OCT4 transcription factor (also known as POU5F1), NBFR-FF-bESCs were permeabilized and blocked in Perm/Wash Buffer supplemented with 2% v/v of NDS for 15 min at room temperature. Cells were then incubated with anti-POU5F1 (1:500; sc-8628, Santa Cruz Biotechnology) primary antibody for 1 h at room temperature and washed with Perm/Wash Buffer. Secondary antibody incubation was performed for 30 min at room temperature with donkey anti-goat IgG Alexa Fluor 488 antibody (1:500; A-11055, Invitrogen) protected from light. Cells were kept at 4 °C until flow cytometry analysis was performed.

Cell-cycle analysis was performed fixing NBFR-FF-bESCs with ice-cold 70% ethanol and stained using Propidium Iodide/RNase Solution (550825, BD Pharmingen). All data were acquired from two independent NBFR-FF-bESCs lines and analyzed in a FACScan flow cytometer (Becton Dickinson) equipped with a 488 nm excitation laser using the CellQuest Pro Software (Becton Dickinson).

### Embryoid body formation

NBFR-bESCs (#A and #B) were trypsinized and separated from the MEF feeders by incubating the cell suspension in a cell culture dish for 1 h in NBFR medium supplemented with 10 μM ROCKi at 37 °C, 5% CO_2_. After incubation, supernatant containing NBFR-bESCs depleted of MEF was collected and cells were seeded using the hanging drop method at a concentration of 1000 cells/20 μL drop. After 3 days of culture, formed embryoid bodies were individually transferred to a well of a low attachment 96-well plate (174951, Thermo Scientific) and cultured for up to 3 weeks in DMEM medium (11995-065, Gibco) supplemented with 10–20% fetal bovine serum (FBS, 10437-028, Gibco), 2 mM MEM Non-essential Amino Acid Solution, 1% v/v GlutaMAX Supplement, 100 U/mL Penicillin, 100 μg/mL Streptomycin and 20 ng/mL FGF2. Depending on the experiment, after 2 weeks of embryoid body differentiation (3D) cell aggregates were transferred to a gelatin-coated dish and cultured in 2D for additional 2 weeks.

### Teratoma formation assay

Four different immunodeficient mice (NOD.CB17–Prkd^scid^/J. Stock No: 001303, The Jackson Laboratory) were injected with a male (NBFR #D) or a female (NBFR #Y) bESC line at passage 12 or 14, respectively. Approximately 5 × 10^5^ NBFR-bESCs resuspended in cold growth-factor-reduced Matrigel were injected in each replicate. Twelve weeks after injection, mice were euthanized for tissue collection. Teratomas were fixed using fresh 4% paraformaldehyde in PBS and subjected to H&E staining for histological analysis. Experiments were performed under approval from the UT Southwestern Institutional Animal Care and Use Committee (IACUC, Protocols #2018-102430) and in compliance with the ARRIVE guidelines (http://www.nc3rs.org.uk/page.asp?id=1357).

### Gene expression analysis

Total RNA was extracted from freshly collected cells, blastocysts or tissues using the RNeasy Mini Kit (74104, Qiagen) and DNase treated with the RNase-Free DNase Set (79254, Quiagen). After extraction, RNA was quantified using the Qubit RNA BR Assay Kit (Q10211, Invitrogen) and reverse transcribed using SuperScript III Reverse Transcriptase (18080044, Invitrogen). PCR was performed using the GoTaq Hot Start Green Master Mix (M5122, Promega) followed by agarose gel electrophoresis stained with ethidium bromide. RNA extraction, RNA quantification, cDNA synthesis, and PCR were performed according to the manufacturer’s protocols. The PrimerQuest Tool (Integrated DNA Technologies) was used to design primers (Table S1) spanning an exon-exon junction. Hydroxymethylbilane synthase gene (*HMBS)* was used as a housekeeping control.

Quantitative gene expression analysis was performed by qPCR using PowerUp SYBR Green Master Mix (A25742, Applied Biosystems) in a QuantStudio 3 Real-Time PCR System (A28137, Applied Biosystems) according to manufacturer’s protocol. Two independent samples were run in technical duplicates and relative expression was calculated by the comparative Ct method, normalizing values to the expression of *HMBS* housekeeping gene.

### Sex determination by polymerase chain reaction

NBFR-bESC lines were sexed by PCR targeting the DEAD box helicase 3 gene (*DDX3X/DDX3Y*), which allows discrimination between X and Y chromosomes based on amplicon size^62^. DNA extraction was performed using the DNeasy Blood and Tissue Kit (69504, Qiagen) according to manufacturer’s protocol. DNA was quantified using a NanoDrop 2000C Spectrophotometer (ThermoScientific) and amplified using the Go Taq Hot Start Green Master Mix. Primer sequences are provided in Table S1 and amplicons were observed by agarose gel electrophoresis stained with ethidium bromide. Genomic DNA from female and male bovine fibroblasts was used as control.

### Histone methylation profiling by CUT&RUN

CUT&RUN was performed following a published protocol^63^ with some modifications. Approximately 5 × 10^4^ bESCs per sample were washed twice with 1 mL washing buffer (20 mM HEPES at pH 7.5, 150 mM NaCl, 0.5 mM Spermidine, and 1 × Roche complete protease inhibitor) and resuspended in 60 μL washing buffer. Concanavalin-coated magnetic beads (BP531, Bangs Laboratories), 20 μL per sample, were washed and resuspended in binding buffer (20 mM HEPES–KOH at pH 7.9, 10 mM KCl, 1 mM CaCl2, 1 mM MnCl2), and added to the samples. Then, the samples were incubated at 24 °C for 10 min on Thermomixer at 400 rpm. Sample tubes were placed on the magnetic stand to remove all the liquid. For each sample, 50 μL antibody buffer (washing buffer, 0.1% digitonin, and 2 mM EDTA at pH 8.0) with H3K4me3 (1:100 dilution, C15410003, Diagenode) or H3K27me3 (1:100 dilution, C15410195, Diagenode) was added to the sample tube, and incubated overnight at 4 °C on Thermomixer at 400 rpm. After antibody incubation, the tubes were placed on the magnetic stand to remove the liquid. After washing twice with digitonin buffer (washing buffer containing 0.1% digitonin), samples were resuspended in 50 μL digitonin buffer containing pAG-MNase (dilution 1:20, 15-1016, EpiCypher), and incubated for 10 min at room temperature. After washing samples twice with digitonin buffer, samples were resuspended with 100 μL cold digitonin buffer and equilibrated on ice for 3 min. Targeted digestion was conducted by adding 2 μL CaCl2 (100 mM) on ice for 30 min, and the digestion was stopped by adding 100 μL 2 × stop buffer (340 mM NaCl, 4 mM EGTA at pH 8.0, 20 mM EDTA at pH 8.0, 0.1% digitonin, 100 μg/mL glycogen, 50 μg/mL RNase A). After incubation at 37 °C for 30 min, samples were digested by adding 1.5 μL proteinase K (20 mg/ml), 2 μL 10% SDS and 10 ng carrier RNA (1068337, Qiagen), and then incubated at 65 °C for 30 min. DNA fragments were purified with phenol–chloroform–isoamyl alcohol and washed by ethanol precipitation, and finally dissolved in 25 μL ddH2O.

Library construction was carried out using NEBNext Ultra II DNA Library Prep Kit for Illumina (E7645L, NEB) according to manufacturer instructions. Briefly, after end-repair/A-tailing, ligation, U-excision and post-ligation cleanup with 1.3 × AMPure XP beads (A63881, Beckman), the first round of PCR was performed with KAPA HiFi HotStart Ready Mix (KM2602, KAPA biosystems), universal primer (E6861AA, NEB) and index primer (NEBNext multiplex Oligos kit) with PCR program of 98 °C for 45 s, followed by 98 °C for 15 s and 60 °C for 10 s for 8 cycles, and 72 °C for 1 min. PCR products were purified with 1.3 × AMPure XP beads, and the purified products were subjected for the second round of PCR which was performed using KAPA HiFi HotStart Ready Mix with the same PCR program. The final libraries were purified by 1.3 × AMPure XP beads and used for sequencing in a Nextseq500 system as paired-end 35 bp reads.

After sequencing, raw reads were trimmed with Trim_Galore (v0.4.0) to remove residual adapter sequences and low quality leading and trailing bases (q < 20) with a stringency set to 1. Both paired and unpaired reads were retained if read length after trimming was at least 10 bases. Trimmed reads were aligned to the ARS-UCD1.2 assembly with BWA mem (v0.7.17-r1188) with default settings. Duplicate alignments were removed with PicardTools (v2.8.1). Low quality alignments (q < 5) were removed using SAMtools (v1.7). Genome-wide signal was normalized by reads per kilobase million (RPKM) in 50 bp windows with the DeepTools bamCoverage function (v3.2.0). Normalized signal was then visualized on the UCSC genome browser (smoothingWindow 4; windowingFunction mean; viewLimits 0:10). Peaks were called with epic2 (v0.0.41) with an effective genome fraction set to 0.7, bin size set to 200 bp, and E-value to control genome-wide error rate set to 500. The maximum number of allowed gaps between enriched windows was altered between H3K4me3 (maximum gaps set to 1) and H3K27me3 (maximum gaps set to 5), as H3K4me3 is considered a narrow mark and H3K27me3 is considered broad. Peak sets from replicates were compared with Bedtools intersect (v2.26.0), and peaks that overlapped by at least 1 bp were considered shared. The fraction of reads in peaks (FRiP) score was calculated with the DeepTools (v3.2.0) function plotEnrichment. Genes were considered “marked” by a histone modification if a peak fell within the promoter (2 kb region upstream of a transcription start site). Overlap of peaks with genes was determined by Bedtools intersect (v2.26.0), requiring at least 1 bp of overlap between the genic region and a peak. The genome annotation that was used was Ensembl v99. Only protein-coding genes were considered. Gene sets (Ensembl IDs) were submitted to DAVID (v6.8) for functional enrichment analysis.

For ChIP-Seq data analysis, raw reads were trimmed with Trim_Galore (v0.4.0) to remove residual adapter sequences and low quality leading and trailing bases (q < 20) with a stringency set to 1. Both paired and unpaired reads were retained if read length after trimming was at least 10 bases. Trimmed reads were aligned to the ARS-UCD1.2 assembly with BWA mem (v0.7.17-r1188) with default settings. Duplicate alignments were removed with PicardTools (v2.8.1). Low quality alignments (q < 5) were removed using SAMtools (v1.7). Genome-wide signal was normalized by reads per kilobase million (RPKM) in 50 bp windows with the DeepTools bamCoverage function (v3.2.0). Normalized signal was then visualized on the UCSC genome browser (smoothingWindow 4; windowingFunction mean; viewLimits 0:10).

## Supplementary Information

Supplementary Information.

## Data Availability

The CUT&RUN data that support the findings of this study are openly available at NCBI GEO repository with accession number GSE157053.
