# Supplementary material for: Simplification of culture conditions and feeder-free expansion of bovine embryonic stem cells
Source: Sci Rep. 2021 May 26;11:11045. doi: 10.1038/s41598-021-90422-0 (PMC8155104; doi:10.1038/s41598-021-90422-0)
Supplement: Supplementary file 1 — Supplementary Information. [file 41598_2021_90422_MOESM1_ESM.pdf]

Simplification of culture conditions and feeder-free expansion of bovine embryonic stem cells

Delia Alba Soto[1A], Micaela Navarro[1AB], Canbin Zheng[C], Michelle Halstead [A], Chuan Zhou [A], Carly Guiltinan[A], Jun Wu[C, D], Pablo Juan Ross[A].

[1]Contributed equally to this work.

[A]Department of Animal Science, University of California, 450 Bioletti Way, Davis, CA 95616, USA.

[B]Instituto de Investigaciones Biotecnológicas 'Dr Rodolfo Ugalde', UNSAM-CONICET, Buenos Aires, Argentina.

[C]Department of Molecular Biology, University of Texas Southwestern Medical Center, 5323 Harry Hines Boulevard, Dallas, TX 75390, USA.

[D]Hamon Center for Regenerative Science and Medicine, University of Texas Southwestern Medical Center, 5323 Harry Hines Boulevard, Dallas, TX 75390, USA.

Corresponding author:

Pablo Juan Ross, Ph.D., Department of Animal Science, University of California, 450 Bioletti Way, Davis, CA 95616, USA. Telephone: +1 (530) 771-7225; email: pross@ucdavis.edu

Supplementary Information

Supplementary Data

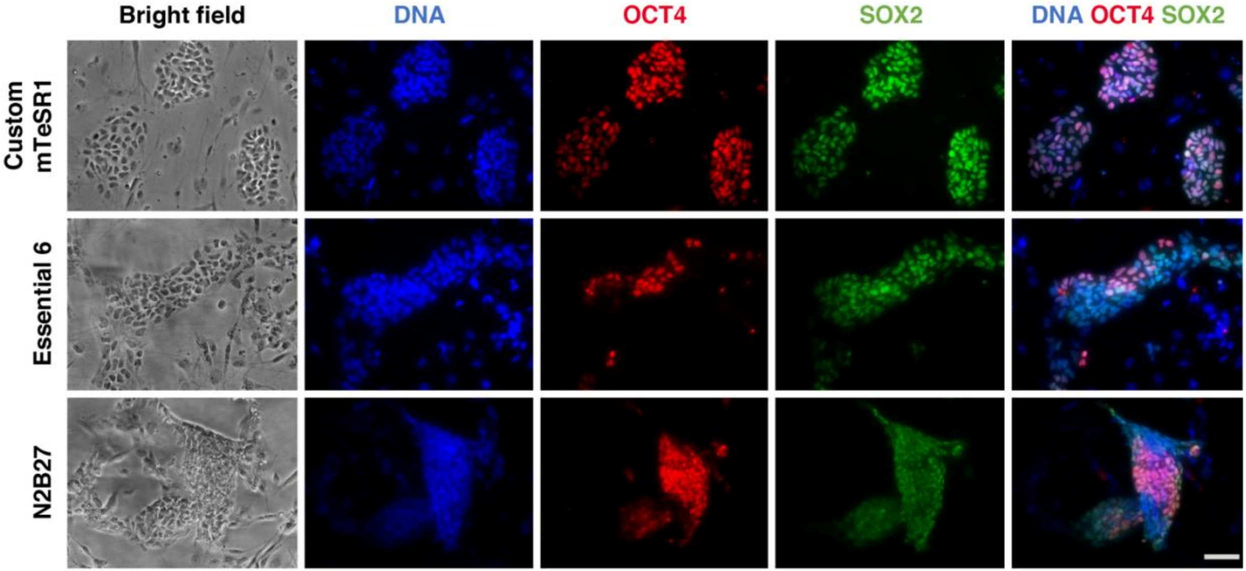

Figure S1. Adaptation of CTFR-bESC to N2B27 and Essential 6 basal media. Immunostaining of pluripotency factors OCT4 and SOX2 in CTFR-bESCs cultured in each base medium alternative. Scale bar 100 µm.

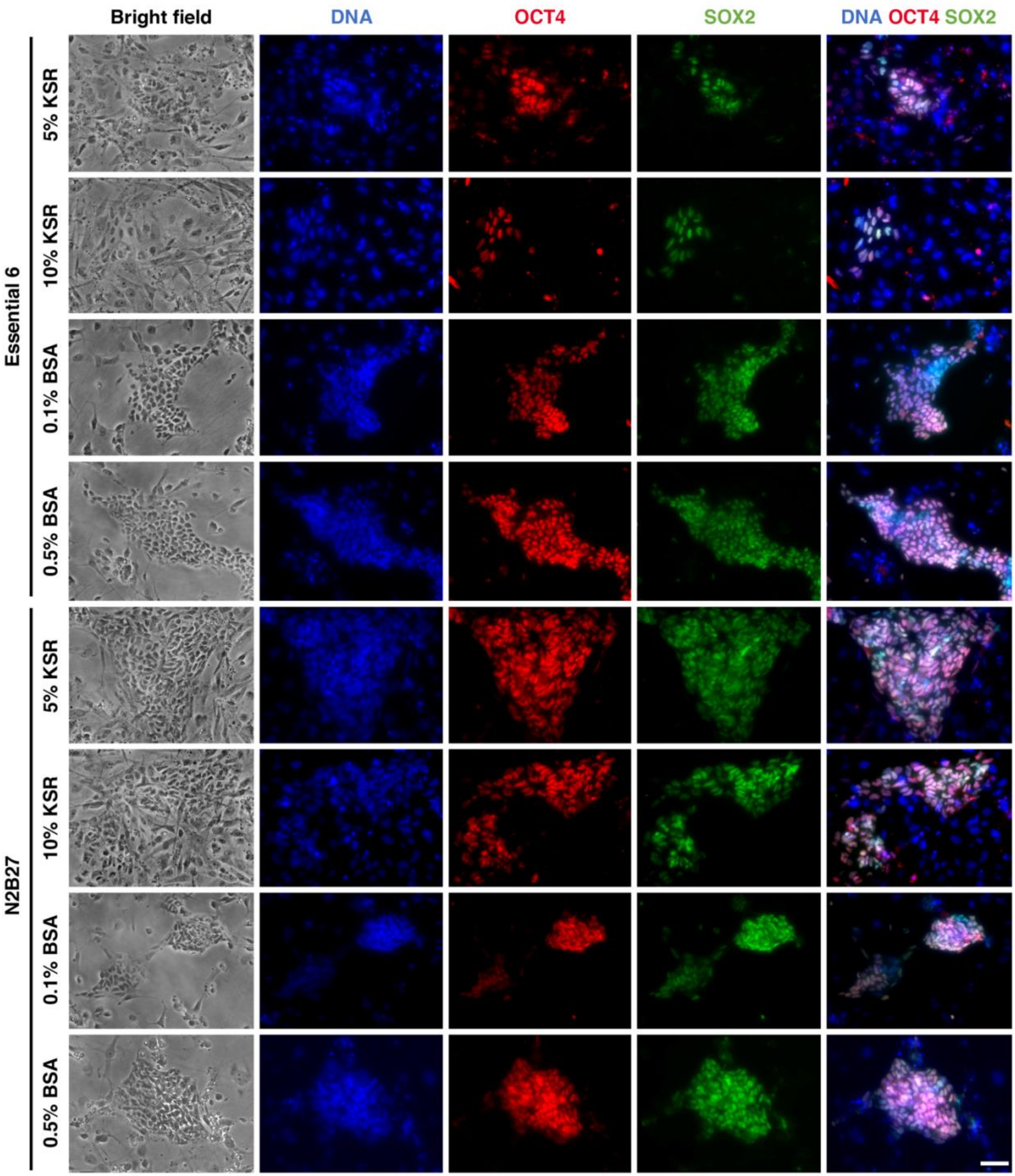

Figure S2. BSA or KSR supplementation of Essential 6 and N2B27 basal media. Immunostaining of pluripotency factors OCT4 and SOX2 in CTFR-bESCs cultured in Essential 6 or N2B27 base medium supplemented with KSR (5% or 10%) or BSA (0.1% or 0.5%). Scale bar 100 µm.

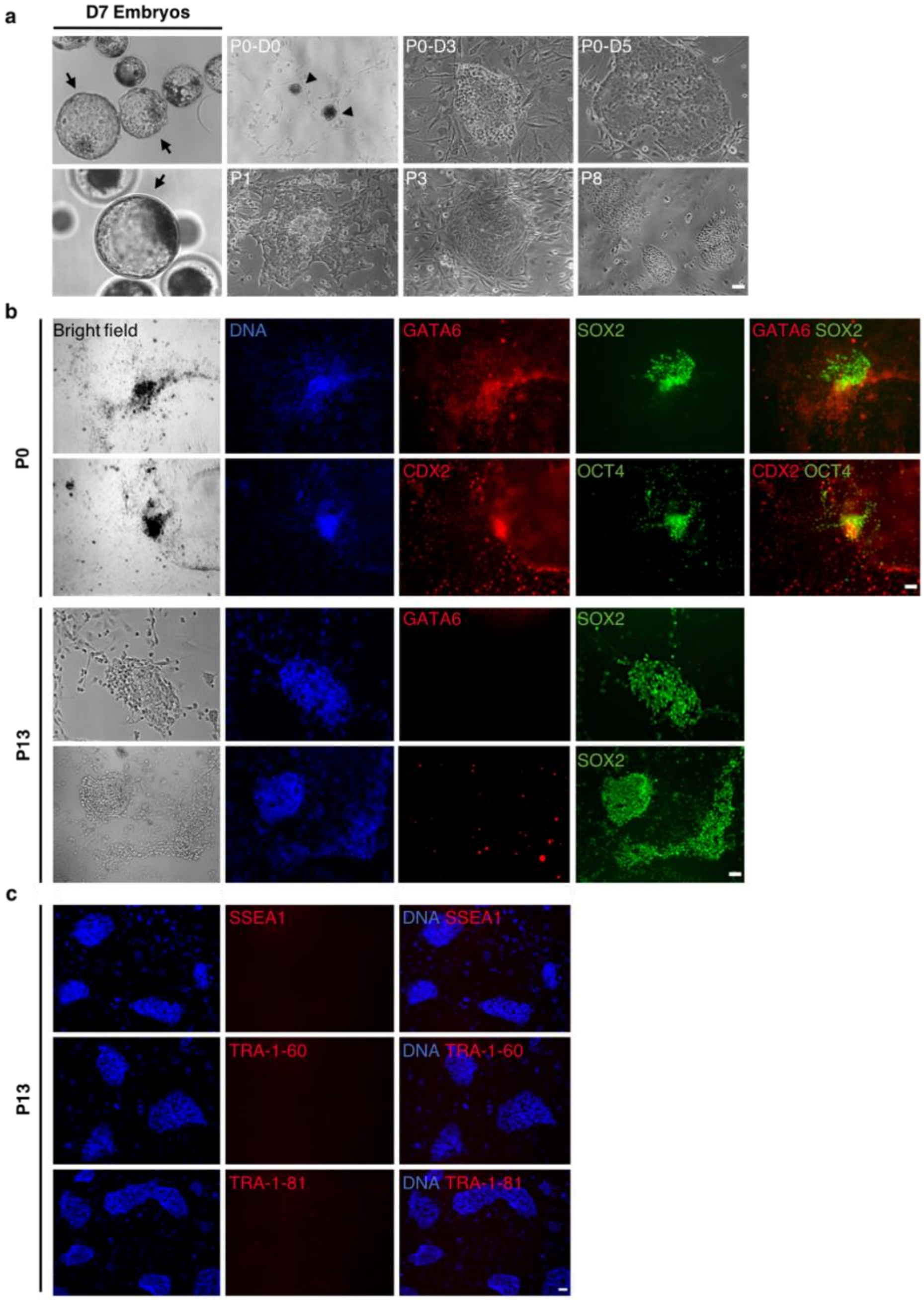

Figure S3. Establishment of NBFR-bESC lines. (a) Bright field images of NBFR-bESCs derived **from isolated ICMs in NBFR conditions**. Representative images of day-7 embryos (arrows) selected for ICMs isolation and ESC derivation, isolated ICMs (arrowheads), outgrowth at passage 0, and typical colony morphology at passages 1, 3, and 8. **(b) Dynamics in the expression of pluripotency** (SOX2, OCT4), trophectoderm (CDX2), and primitive endoderm (GATA6) factors during establishment of NBFR-bESC lines and (**c**) their surface marker profile (SSEA1, TRA-1-60 and TRA-1-81) detected by immunofluorescence staining. Red spots observed in CDX2 picture corresponded to unspecific staining. D: day; P: passage. Scale bars 100 μm.

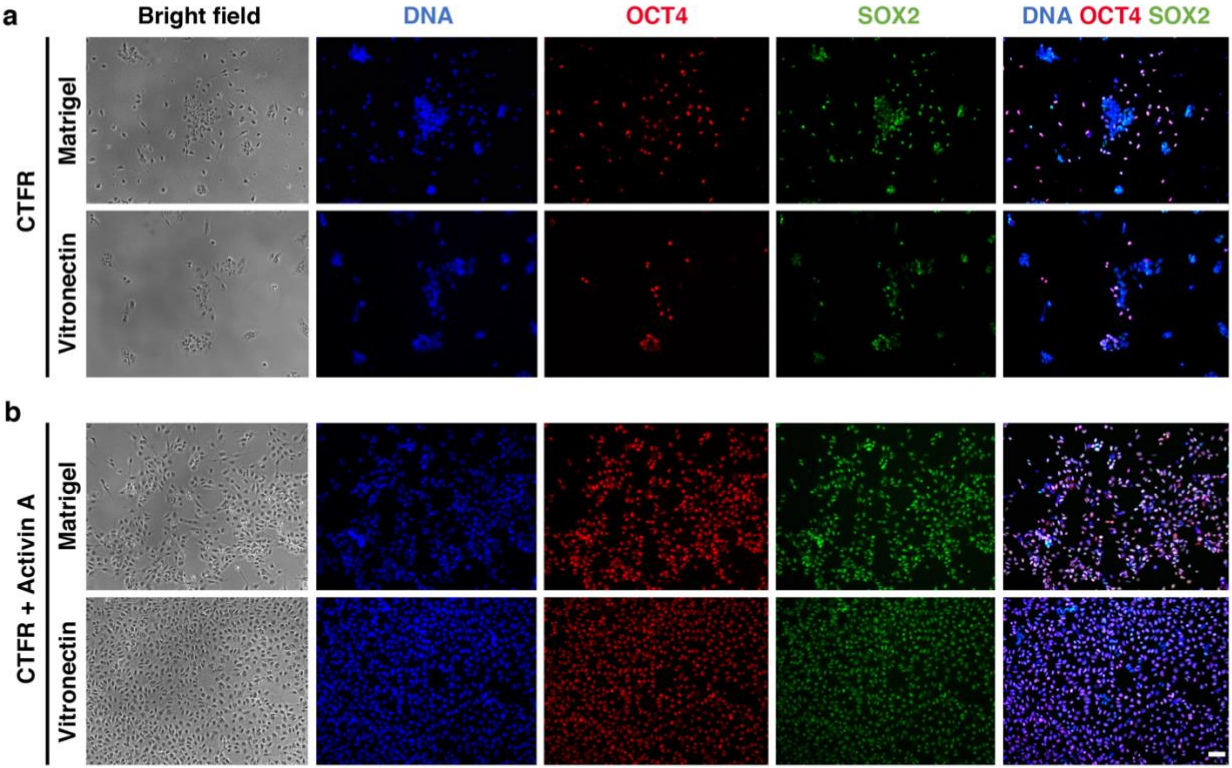

Figure S4. Adaptation of CTFR-bESCs to feeder-free conditions. Immunofluorescence of pluripotency factors OCT4 and SOX2 in CTFR-bESCs adapted to different substrates with or without 20 ng/mL Activin A for 4 passages. Scale bar 100 μm.

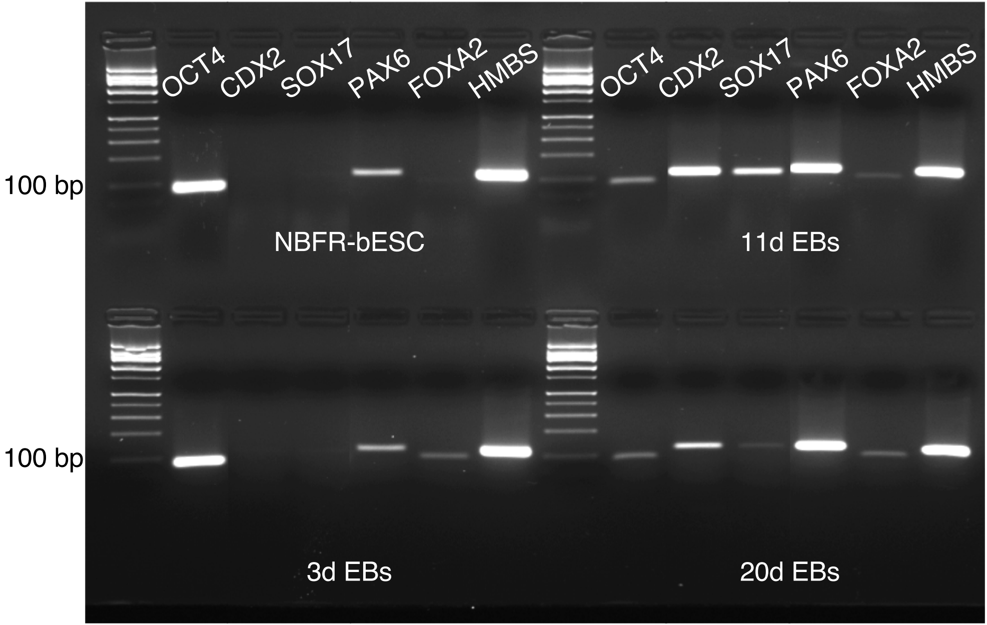

Figure S5. Full-length gel presented in Figure 3b. bp: base pairs; d: days; EBs: embryoid bodies.

Table S1 List of primers used in this study

Gene	Forward Primer Sequence	Reverse Primer Sequence	Tm (°C)	Amplicon length
OCT4	AACGAGAATCTGCAGGAGATATG	TCTCACTCGGTTCTCGATACT	62	87
NANOG	CAGCTACAAGCAGGTGAAGA	CTATTCCTCGGCCAGTTGTT	62	84
SOX2	CATTAACGGCACACTGCCCC	TGAAAATGTCTCCCCCGCCC	62	76
SOX17	AAGATGCTGGGCAAGTCG	CGGTACTTGTAGTTGGGATGG	62	116
CDX2	TGGGCAGCCAAGTGAAA	CTTTCCTCCGGATGGTGATATAG	62	120
MEOX1	GGAGAATTCAGACAACCAGGAG	TGAGCAAACTCAGCTTCGAG	62	117
FOXA2	CCCTTCTCCATCAACAACCT	GTAGGCCTTGAGGTCCATTT	62	93
PAX6	CCCTGGAGAAAGAGTTTGAGAG	TCCATTTGGCCCTTCGATTAG	62	126
DDX3	AGGAAGCCAGGAAAGTAA	CATCCACGTTCTAAGTCT	58	184 and 208
HMBS	CTTCACCATTGGAGCTGTCT	TAGTTCCTACCACACTCTTCTCT	62	116

Table S2 Summary of reads, alignments and peaks called for each sample

Histone mark	Sample	Raw reads	Aligned reads	Informative reads	Peaks	FRiP score	Peaks detected in other replicate
H3K4me3	ESC rep. 1	53,571,600	46,743,154	34,418,223	26,048	32.21	21,636 (83%)
H3K4me3	ESC rep. 2	48,298,890	42,082,536	30,904,062	23,986	32.12	21,511 (90%)
H3K27me3	ESC rep. 1	42,716,486	36,372,661	26,109,361	24,430	43.65	22,232 (91%)
H3K27me3	ESC rep. 2	66,047,384	56,632,811	41,982,903	21,244	45.13	17,691 (83%)
Average		52,658,590	45,457,791	33,353,637			
